# Baculovirus-Based Biocontrol: Synergistic and Antagonistic Interactions of PxGV, PxNPV, SeMNPV, and SfMNPV in Integrative Pest Management

**DOI:** 10.3390/v17081077

**Published:** 2025-08-02

**Authors:** Alberto Margarito García-Munguía, Carlos Alberto García-Munguía, Paloma Lucía Guerra-Ávila, Estefany Alejandra Sánchez-Mendoza, Fabián Alejandro Rubalcava-Castillo, Argelia García-Munguía, María Reyna Robles-López, Luis Fernando Cisneros-Guzmán, María Guadalupe Martínez-Alba, Ernesto Olvera-Gonzalez, Raúl René Robles-de la Torre, Otilio García-Munguía

**Affiliations:** 1 Centro de Investigación en Biotecnología Aplicada (CIBA), Instituto Politécnico Nacional (IPN), Ex-Hacienda San Juan Molino Carretera Estatal Tecuexcomac-Tepetitla Km. 1.5, Tlaxcala 90700, Mexico; alberto.garcia@edu.uaa.mx (A.M.G.-M.); munguia.ca@ugto.mx (C.A.G.-M.); mrobles@ipn.mx (M.R.R.-L.); 2 Centro de Ciencias Agropecuarias, Universidad Autónoma de Aguascalientes, Avenida Universidad # 940, Col. Ciudad Universitaria, Aguascalientes 20131, Mexico; paloma.guerra@edu.uaa.mx (P.L.G.-Á.); estefany.sanchez@edu.uaa.mx (E.A.S.-M.); fabian.rubalcava@edu.uaa.mx (F.A.R.-C.); argy_2511@hotmail.com (A.G.-M.); fernando.cisneros@edu.uaa.mx (L.F.C.-G.); 3 Departamento de Veterinaria y Zootecnia, Universidad de Guanajuato, Carretera Irapuato—Silao, Km. 9, Guanajuato 36500, Mexico; 4 Departamento de Agronomía, Universidad de Guanajuato, Carretera Irapuato—Silao Km. 9, El Copal, Complejo 2 de la DICIVA, Irapuato 36500, Mexico; 5 Departamento de Ecología y Recursos Naturales, Centro Universitario de la Costa Sur, Universidad de Guadalajara, Av. Independencia Nacional 151, Autlán de Navarro 48900, Mexico; 6 Tecnológico Nacional de México-Instituto Tecnológico de Pabellón de Arteaga, Carretera a la estación de Rincón de Romos, Km. 1, Pabellón de Arteaga 20670, Mexico; mtzalba@live.com.mx (M.G.M.-A.); e.olvera.itp@gmail.com (E.O.-G.)

**Keywords:** diamondback moth, beet armyworm, fall armyworm, vegetable, corn, synthetic insecticide, cultivation, entomopathogenic virus, biological pest control

## Abstract

The use of chemical pesticides in agriculture has led to the development of resistant pest populations, posing a challenge to long-term pest management. This review aims to evaluate the scientific literature on the individual and combined use of baculoviruses with conventional chemical and biological insecticides to combat *Plutella xylostella*, *Spodoptera exigua*, and *Spodoptera frugiperda* in broccoli, tomato, and maize crops. Notable findings include that both individual *Plutella xylostella* nucleopolyhedrovirus (PxNPV) and the combination of *Plutella xylostella* granulovirus (PxGV) and azadirachtin at a low dose effectively control *Plutella xylostella;* both combinations of *Spodoptera exigua* multiple nucleopolyhedrovirus (SeMNPV) with emamectin benzoate and chlorfenapyr reduced resistance in *Spodoptera exigua* and increased the efficacy of the insecticides; and the combination of *Spodoptera frugiperda* nucleopolyhedrovirus (SfMNPV) and spinetoram is effective against *Spodoptera frugiperda.* Integrating baculoviruses into pest management strategies offers a promising approach to mitigate the adverse effects of chemical pesticides, such as resistance development, health risks, and environmental damage. However, there remains a broad spectrum of research opportunities regarding the use of baculoviruses in agriculture.

## 1. Introduction

Broccoli (*Brassica oleracea*), tomato (*Solanum lycopersicum*), and maize (*Zea mays*) are agronomic keystones across global production systems [1,2,3]. Their high nutritional profiles, economic value, and extensive cultivation make them indispensable for food security, particularly in regions where agriculture represents a primary economic activity [4]. These crops also serve as vital sources of employment, especially in rural communities [5].

Yet, their productive potential is persistently threatened by high-impact insect pests. Among the most damaging are *Plutella xylostella* (broccoli) [6], *Spodoptera exigua* (tomato) [7], and *Spodoptera frugiperda* (maize) [8]. These species demonstrate extraordinary ecological plasticity and rapid adaptation to management interventions [9,10]. Alarmingly, they have evolved resistance to several commonly used insecticides, rendering many conventional control strategies increasingly ineffective [11].

In response to these escalating challenges, integrated pest management (IPM) has emerged as the preferred paradigm for sustainable pest control [12]. IPM promotes a multi-tiered approach by integrating agronomic practices, monitoring tools, economic threshold models, and biological control agents to reduce pest populations while minimizing environmental harm [13]. By steering away from sole reliance on synthetic insecticides, IPM fosters ecological resilience, safeguards non-target organisms, and delays resistance development [14].

Within this strategic framework, baculoviruses have gained considerable attention as powerful biological control tools [15]. Their exceptional host specificity, environmental safety, and proven field performance align seamlessly with the goals of sustainable agriculture [12]. Belonging to the family *Baculoviridae*, these viruses are classified into four genera *Alphabaculovirus*, *Betabaculovirus*, *Gammabaculovirus*, and *Deltabaculovirus* based on host range and virion morphology [16]. *Alphabaculovirus* and *Betabaculovirus*, which include nucleopolyhedroviruses (NPVs) and granuloviruses (GVs), respectively, primarily target lepidopterans pests [17], while the less-studied *Gammabaculovirus* and *Deltabaculovirus* infect hymenopterans and dipterans [18,19].

Baculoviruses possess a circular, double-stranded DNA genome [20] and are renowned for their ability to form inclusion bodies—polyhedral in NPV and granular in GVs—that enhance their persistence in the environment [21]. This biological durability, combined with their host specificity, minimizes off-target effects and environmental disruption [15]. They infect the larval stages of pests, leading to systemic infection and eventual death, thereby exerting population-level suppression without adverse impacts on beneficial organisms [22].

In agricultural contexts, baculoviruses have been successfully used to control various pests [23]. *Alphabaculovirus spofrugiperdae*, commonly called *Spodoptera frugiperda* multiple nucleopolyhedrovirus (SfMNPV), has shown efficacy in reducing *Spodoptera frugiperda* populations in maize [24,25,26]. Similarly, *Alphabaculovirus spexiguae* (*Spodoptera exigua* multiple nucleopolyhedrovirus) (SeMNPV), has been successfully deployed in tomato. The so-called *Plutella xylostella* nucleopolyhedrovirus (PxNPV), now considered a variant of *Alphabaculovirus aucalifornicae*, has been used in broccoli systems, achieving significant pest suppression [27,28,29,30]. These results reinforce the versatility and cross-systems applicability of baculoviruses under varying agroecological conditions.

The present study conducts a comparative evaluation of these baculoviruses across the three cropping systems, focusing on their effectiveness relative to conventional chemical pesticides. It further explores the biological and ecological factors that influence success rates and transferability of these control agents across crop contexts. Through a critical review of recent applications, this work elucidates the untapped potential of baculoviruses and proposes pathways for optimizing their integration into next-generation IPM frameworks.

## 2. Information Retrieval Strategy

A comprehensive review of scientific literature was conducted using the literature review method. This research involved several stages: identification, selection, and assessment of study eligibility. The choice of this method was based on its systematic, replicable, and comprehensive nature, which aims to minimize bias through precise and meticulous bibliographic searches, ensuring a transparent and rigorous scientific process. Furthermore, it provides a detailed account of the procedures followed by the researchers. In this study, we specifically focused on works analyzing the impact of baculovirus application as a biological control agent. This review offers a thorough and up-to-date overview of the latest research in this emerging field.

### 2.1. Selection and Collection of Studies

We followed the step-by-step framework outlined by [31] which encompasses five distinct stages, as seen in Table 1.

To develop the research question for this study, an adaptation of the PICO strategy developed by [31] was followed. This approach uses the acronym PICO to represent the Problem (P), the Intervention (I), the Comparison (C), and the Outcome (O). In this context, “P” refers to broccoli crops affected by *Plutella xylostella*, tomato crops affected by *Spodoptera exigua*, and maize crops affected by *Spodoptera frugiperda*; “I” denotes the application of specific baculovirus for each pest; “C” refers to the use of conventional chemical insecticides; and “O” alludes to the reduction in pest populations and improvement in crop health and yield.

Therefore, the research question for this study was formulated as follows: “To what extent does the application of specific baculovirus reduce the population of *Plutella xylostella* in broccoli crops, *Spodoptera exigua* in tomato crops, and *Spodoptera frugiperda* in maize crops, compared to the use of conventional chemical insecticides?” To address this question comprehensively, a search of scientific articles was conducted in February 2025.

#### 2.1.1. Identification

The article search for this review was conducted using the electronic databases Scopus and EndNote Software. Initially, searches were classified by crop type. For broccoli crops, the selected descriptors were “baculovirus” AND “*Plutella xylostella*”, yielding 62 results. For tomato crops, the terms “baculovirus” AND “tomato” AND “*Spodoptera*” were used, resulting in 14 outcomes. Lastly, for maize crops, the search terms “baculovirus” AND “maize” AND “*Spodoptera frugiperda*” were employed, obtaining 27 results. Regarding insecticides, the research terms “insecticide” AND “*Plutella xylostella*” AND “broccoli” yielded 127 results. For tomato crops, the terms “insecticide” AND “tomato” AND “*Spodoptera exigua*” resulted in 6 outcomes. Finally, for maize crops, the search terms “insecticide” AND “maize” AND “*Spodoptera frugiperda*” yielded 336 results.

In total, 675 articles were identified from Scopus and 572 from EndNote. The search results were saved in the respective databases for further analysis. The inclusion criteria for the studies were as follows: the article included baculovirus as a biological control agent in crops. Articles with relevant titles and abstracts related to the study topic were included. Conversely, the exclusion criteria were the following: the article did not focus on pest population reduction, crop health improvement, and/or yield enhancement; it was a conference review; it was a letter, erratum, data article, or note.

#### 2.1.2. Screening

The authors assessed the titles and abstracts of the 258 identified articles. To proceed to the next stage, the articles needed to meet specific criteria: they had to discuss the use of baculovirus as biological agents in broccoli, tomato, and maize crops; provide detailed mechanisms of action of baculoviruses; include the use of insecticides in these crops; and explore improvements in pest population reduction, crop health, and/or yield. Out of the 258 initially selected articles, only 165 met these criteria. These articles were then exported to the personal EndNote library for further analysis.

#### 2.1.3. Eligibility

The authors thoroughly examined the full text of the 165 selected articles. Articles were excluded if they met any of the following criteria: the full article was not available, the article did not involve the application of baculovirus, or the article did not pertain to agricultural applications. In this phase, 53 articles were excluded, leaving 112 articles as the basis for this review.

#### 2.1.4. Data Analysis

**Figure 1:**
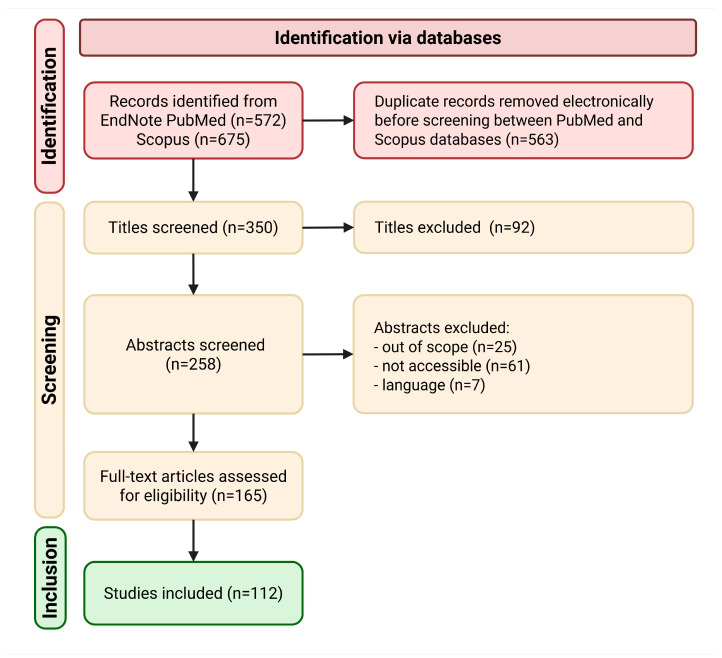
Methodology flow diagram.

The 112 studies that successfully passed the Identification, Screening, and Eligibility phases were exported to Excel for further analysis and consolidation of findings. A structured data extraction form was created to gather pertinent information from each selected study, including details such as the author(s), publication year, crop, pest, and treatment. Based on the synthesized findings, potential future perspectives for pest management in the crops were identified and discussed, with a focus on innovative and sustainable approaches. The process followed is illustrated in Figure 1.

## 3. Results

### 3.1. Economic Impact

Broccoli, tomato, and maize are essential crops in the global agricultural economy, valued for their profitability and employment generation [30,32,33,34,35,36]. According to FAO data, annual global production reaches approximately 22.8 million tons for broccoli and cauliflower [1], 180 million tons for tomatoes [2], and 1.16 billion tones for maize [3]. Despite their economic significance, these crops face serious threats from pests that compromise yield and market stability. *Plutella xylostella*, a major pest in broccoli cultivation, causes losses exceeding four to five billion dollars annually, with severe infestations leading to crop losses of up to 90% [6,37]. Tomato crops are particularly vulnerable to *Spodoptera exigua*, which can result in yield reductions of 80–100% if unmanaged [5,7]. Similarly, maize production is heavily affected by *Spodoptera frugiperda*, responsible for annual yield losses of approximately 20–30%, primarily due to maize stem borers [8,38]. These pests not only reduce crop quality and commercial value but also disrupt market stability [5,39,40,41,42]. Additionally, the increasing resistance of insect pests to pesticides exacerbates control costs, leading to global economic losses that may amount to billions of dollars [11,43].

### 3.2. Impact and Management of Infestations in Crops

The management of broccoli, tomato, and maize crops is highly dependent on integrated pest control strategies to mitigate economic losses and sustain agriculture [44]. *Plutella xylostella* in broccoli, *Spodoptera exigua* in tomatoes, and *Spodoptera frugiperda* in maize present severe challenges due to their feeding habits, which compromise yield and quality [5].

Integrated pest management (IPM) combines cultural, biological, and chemical approaches to maintain pest populations at manageable levels. Cultural control methods, such as crop rotation and continuous monitoring, help reduce infestation risks productivity [45]. Chemical insecticides, including carbamates, organophosphates, pyrethroids, neonicotinoids, avermectins, milbemycins, oxadiazines, diamides, and others, remain widely employed due to their broad-spectrum efficacy and diverse modes of action. In parallel, natural insecticides such as spinosyns, *Bacillus thuringiensis* (Bt), granuloviruses, and nucleopolyhedroviruses continue to play a vital role in sustainable pest management strategies, offering targeted control with reduced environmental impact [11]. However, the continuous and repeated use of the same chemical products has led to resistance in insect populations, which is subsequently inherited by their offspring [46]. Biological pesticides, such as *Bacillus thuringiensis* (Bt) toxins, offer an effective solution against *S. exigua*, disrupting larval development without harming non-target species [47,48]. Similarly, selective insecticides like chlorantraniliprole and emamectin benzoate are utilized against *S. frugiperda*, although resistance development remains a concern [44]. Diversified pest management strategies, integrating chemical control with biological and ecological approaches, are essential to maintaining crop viability while preventing resistance escalation [49], as seen in Table 2.

### 3.3. Mechanisms of Resistance: Metabolic Adaptations and Target-Site Mutations

Insecticide resistance is primarily driven by metabolic adaptations and target-site mutations. In *Plutella xylostella*, studies indicate that 56% of resistance cases are attributed to target-site mutations, while 44% result from metabolic resistance mechanisms.

Target-site mutations, including point mutations, amino acid substitutions, and reduced insecticide binding affinity, represent a major resistance mechanism. These genetic alterations decrease the efficacy of insecticides and promote the survival of resistant individuals within exposed populations. For example, diamide insecticides, widely used in pest management, are associated with target-site mutations, contributing to the reported 56% resistance [11]. Additionally, chlorantraniliprole influences resistance through the activation of ryanodine receptors, indirectly regulating cytochrome P450 monooxygenase activity, which accounts for a portion of the 44% metabolic resistance [11]. This inheritable resistance diminishes the efficacy of pesticides, compelling farmers to seek more sustainable alternatives [114]. Several mutations have been linked to insecticide resistance in *P. xylostella*: the I4790M and G4946E mutations in the *RyR* gene confer resistance to diamide insecticides. A three-amino-acid deletion in the transmembrane domain of the nAChR α6 subunit provides high-level resistance to spinosad. *I1042M* mutation in the *CHS1* gene is associated with resistance to benzoylurea (BPU) insecticides, including diflubenzuron, flufenoxuron, lufenuron, and flucycloxuron [115].

Reactive oxygen species (ROS) activate the *CncC/Maf* signaling pathway, enhancing *GST* gene expression following insecticide exposure. *CncC/Maf* functions as a key regulator of detoxification genes involved in insecticide resistance, particularly in *Spodoptera exigua*. Thus, *CncC/Maf* inhibitors could serve as broad-spectrum synergists to mitigate resistance in pest populations [116].

Moreover, insecticide-resistant populations exhibit increased expression of cytochrome P450 genes involved in drug metabolism, carbon metabolism, oxidative phosphorylation, fatty acid metabolism, and protein processing. Specifically, the genes *CYP405D1*, *CYP6AB269*, and *CYP4AU1* are significantly upregulated in tetraniliprole-resistant insects [117].

A study on *Spodoptera frugiperda* identified several mutations that contribute to resistance development, including the following: F290C (*ace-1* gene), I1040T/V (*CHSA* gene), A309T (*GluCl* gene), and I4790T/V (*RyR* gene) [115].

Furthermore, the L-aminoacylase-encoding gene, *SfruACY*, upregulated in *Spodoptera frugiperda* larvae feeding on maize, lowers the acid-amino acid conjugates (FACs) content in oral secretions. This process potentially weakens plant defense responses, facilitating larvae growth [118].

The enzyme *Glutathione S-transferase (SfGSTe1)* enhances *Spodoptera frugiperda* resistance to chlorantraniliprole, as its expression is significantly upregulated following exposure. Moreover, SfGSTe1 mRNA stability is regulated by miR-10-5p, which prevents mRNA degradation, thereby reinforcing insecticide resistance [119].

### 3.4. Evaluating Baculoviruses as a Sustainable Strategy for Integrated Pest Management (IPM)

Baculoviruses are highly specific and represent a complementary tool, particularly when resistance to other methods is present, making them a valuable tool in modern pest management [15,120]. Baculoviruses and chemical insecticides employ distinct mechanisms of action against pest insects, which directly affect their efficacy and specificity [121].

Differences in target sites: Chemical insecticides typically act on neuronal receptors, ion channels, or key enzymes in the insect’s metabolism, leading to rapid paralysis and death [122]. In contrast, baculoviruses infect midgut cells, replicate within the host, and cause a systemic infection, ultimately leading to insect mortality through liquefaction—a hallmark of viral infection [120].

Control of pesticide-resistant insects: Insects that have developed resistance to chemical pesticides may remain susceptible to baculoviruses, as these viruses operate entirely different mechanisms [123]. Furthermore, baculoviruses exhibit high specificity, minimizing impact on beneficial organisms and supporting their application in sustainable biological control programs [13].

#### 3.4.1. Overview of the Baculoviruses

The Baculoviridae family is classified into four major genera based on viral particle morphology and host range: *Alphabaculovirus*, *Betabaculovirus*, *Gammabaculovirus*, and *Deltabaculovirus* [16]. Within this family, baculoviruses are further divided into two primary genera: nucleopolyhedrovirus (NPV) and granulovirus (GV). NPVs form large polyhedral inclusion bodies, each containing multiple virions, whereas GVs produce smaller granular inclusion bodies, typically encapsulating a single virion [21].

The occlusion-derived viruses (ODVs) are encapsulated within crystalline structures called occlusion bodies (OBs). These bodies form inside the nucleus of infected cells and are released when the host larvae disintegrate. These bodies may be polyhedral, containing multiple ODVs, or granular, containing only one. They are primarily composed of proteins, such as polyhedrin or granulin [124]. Baculoviruses contain double-stranded DNA (dsDNA) as their genetic material. Their genome is circular and supercoiled, ranging from 80 to 180 kilobases (kb) in size [17]. This organization enables them to encode many proteins essential for their life cycle and replication within host cells.

#### 3.4.2. General Factors of Molecular Biology of Baculoviruses

Baculoviruses are specific viruses that infect only certain pest insects, without affecting other beneficial organisms, plants, or mammals [15]. They possess several specific genes and proteins that facilitate their ability to infect the host [123]. One such example is P74 (PIF0), an essential *per os* infectivity factor (PIF) for oral infection in insects [125]. P74 binds to the midgut cells of the insect, facilitating viral entry [126]. There are at least 10 *per os* infectivity factors (pif) genes encoded by *Autographa californica* multiple nucleopolyhedrovirus (AcMNPV), including PIF1 (Ac119), PIF2 (Ac22), PIF3 (Ac115), PIF4 (Ac96), PIF5 (Ac148), PIF6 (Ac68), PIF7 (Ac110), PIF8 (Ac83), and PIF9 (Ac108), which work in conjunction with P74-PIF0 (Ac138) to mediate the binding and entry of the virus into midgut cells. Orthologs of PIF genes (except PIF9) are present in all baculovirus genomes [127]. Enhancins, proteins that enhance viral infectivity, achieve this by degrading the peritrophic membrane of the insect midgut, enabling the virus to reach the epithelial cells [123]. GP64 and F proteins facilitate viral entry into insect cells through their role in envelope fusion, with GP64 found in *alphabaculoviruses* and F protein in both *alphabaculoviruses* and *betabaculoviruses* [128,129]. These genes and proteins enable baculoviruses to infect their host precisely without affecting other organisms.

Ac145 and Ac150 are potential PIF proteins. These two genes encode small proteins (≈9 and 11 kDa, respectively) that are related to each other (23% amino acid sequence identity) and are located in the ODV envelopes. Ac145 and Ac150 encode a domain believed to bind chitin. Occluded virions lacking Ac150 were significantly less virulent when administered *per os* compared to the wild-type virus in larvae of *Heliothis virescens*, *Spodoptera exigua*, and *Tichiplusia ni*. Evidence suggested that the mutant had a reduced capacity to establish primary infections in midgut cells [130].

#### 3.4.3. Specific Findings on Baculoviruses in *Plutella xylostella*

A genomic study of *P. xylostella* identified five Short Interspersed Nuclear Elements (SINEs): *PxSE1*, *PxSE2*, and *PxSE3*, which are tRNA-derived retrotransposons, and *PxSE4* and *PxSE5*, derived from 5S RNA retrotransposons. The insertion of these SINEs into key regions of the viral or host genome may influence baculovirus replication efficiency. Notably, the preference of *PxSINEs* for insertion or accumulation within intronic regions suggests that these elements contribute to structural variation in introns. Furthermore, identifying *PxSE1*-like elements in baculoviruses and their lepidopteran host provides evidence of horizontal gene transfer, likely facilitated by host–parasite interactions [131].

Baculoviruses infecting *Plutella xylostella* belong to both major genera: nucleopolyhedrovirus (NPV) and granulovirus (GV).

Regarding *Plutella xylostella* nucleopolyhedrovirus (PxNPV), the strain PxNPV_LBIV-11, isolated from *P. xylostella* larvae, has been identified as an *alphabaculovirus*. A morphological characterization using SEM microscopy revealed that its polyhedra are quasi-cubic and contain multiple virions (220 × 25 nm) within each enveloped infective unit, characterized by the presence of the *gp64* gene. The significance of *gp64* in the control of the *diamondback moth* (*P. xylostella*) lies in its role in enhancing baculovirus infectivity and propagation within the host [132].

Additionally, baculoviruses infecting *P. xylostella* have been reported to contain the Ac137 protein, which consists of 94 amino acids with a molecular weight of 10.3 kDa [127]. This protein is homologous to the p10 protein found in most Group I and II NPVs and several GVs, sometimes occurring in multiple copies. The p10 gene was originally identified as a highly expressed very late gene [133]. Originally identified as a highly expressed late-stage gene, three orthologs of *p10* (*px002*, *px021*, and *px050*) have been detected in the PxGV genome. These orthologs are predicted to share a structural resemblance to reovirus protein [127]. A plausible hypothesis suggests that the *p10* gene and its protein product contribute to the stability and persistence of baculoviruses in culture.

#### 3.4.4. Specific Findings on *Spodoptera exigua* nucleopolyhedrovirus

At the molecular level, the genome of SeMNPV spans approximately 135,764 base pairs (bp) and encodes 136 open reading frames (ORFs), which govern essential proteins involved in viral replication and pathogenicity [134].

One notable protein is Protein Tyrosine Phosphatase-2 (PTP2). All Group I *alphabaculoviruses* encode an ortholog of protein tyrosine phosphatase [127,135]. These viruses are divided into two major lineages, with most members of one lineage carrying an additional *ptp* gene, designated *ptp-2* [127,129]. In SeMNPV, PTP2 induces mild apoptosis when transiently expressed in *Spodoptera frugiperda* Sf21 cells and infected larvae. Deletion of *ptp2* results in reduced production of viral occlusion bodies [136].

Additionally, ribonucleotide reductase (RR), a heterodimer composed of large and small subunits (*RR1* and *RR2*, respectively), plays a crucial role in baculovirus biology [137]. Phylogenetic analyses suggest that baculovirus acquired the *RR1* gene through two distinct capture events from a bacterial source for *Orgyia pseudotsugata* multicapsid polyhedrosis virus (OpMNPV) and *Lymantria dispar* multicapsid nuclear polyhedrosis virus (LdMNPV), while the lineage associated with SeMNPV appears to have originated independently rather than through gene duplication [137].

#### 3.4.5. Specific Findings of *Spodoptera frugiperda* nucleopolyhedrovirus SfMNPV for Mitigating *Spodoptera frugiperda* in Maize

All baculoviruses are known to influence gene regulation in their insect host; however, *Spodoptera frugiperda* nucleopolyhedrovirus strain Ar (SfMNPV-Ar) is particularly notable as an exotic and highly virulent variant that specifically affects corn earworm populations in Mexico. Once infection with SfMNPV-Ar is initiated, its regulatory impact on host gene expression becomes pronounced, contributing to its effectiveness as a biocontrol agent. This regulation is linked to various cellular processes, including cytoskeletal organization, cellular metabolism, oxidative stress response, apoptosis, protein folding, translation, and ribosomal structure. Understanding these mechanisms provides critical insight into how the virus modulates host metabolism during the early stages of infection [138].

Given these promising findings, optimizing the timing of baculovirus application is essential for effective pest control. Reports indicate that high humidity and reduced sunlight hours in September create favorable conditions for NPV proliferation and infection in fall armyworm populations. This highlights the potential of NPVs as sustainable alternatives for controlling maize pests [139].

### 3.5. Interactions Between Baculoviruses and Insecticides

The combined use of viral agents with chemical or biological compounds has emerged as a key strategy to optimize pest control. In this context, interactions between baculoviruses and insecticides can be primarily classified as synergistic or antagonistic.

A synergistic interaction occurs when the combined efficacy of two agents exceeds the sum of their individual effects, resulting in higher mortality, reduced application doses, or enhanced horizontal transmission of the virus [140].

In contrast, an antagonistic interaction arises when one component interferes negatively with the action of the other, diminishing treatment efficacy, accelerating host death before viral replication is complete, or even inactivating the viral particles [140].

Relevant examples of both categories are detailed below.

#### 3.5.1. Synergistic Interactions

Numerous studies have documented synergistic interactions between baculoviruses and both natural and synthetic insecticides. For instance, the combination of PxGV and azadirachtin (AZA) (CAS number 11141-17-6), a biologically active compound derived from neem oil (*Azadiracha indica*), has demonstrated potent efficacy and a synergistic effect (Combined Total Concentration, CTC > 20) at low doses in controlling the diamondback moth [141]. Similarly, in resistant populations of *P. xylostella*, mixtures of PxGV with indoxacarb or fipronil have demonstrated compatibility and synergism [142].

For SeMNPV, combining it with juvenile hormone analogs such as methoprene or pyriproxyfen increases the production of occlusion bodies (OBs) in infected larvae, thereby enhancing viral efficacy and persistence in the field [143]. Moreover, the addition of a low dose of SeMNPV enhances the effectiveness of compounds such as stilbenes, emamectin benzoate, chlorantraniliprole, and spinetoram by reducing the median lethal dose (LD_50_) under both laboratory and field conditions [29]. Flufenoxuron, a chitin synthesis inhibitor, has been reported to enhance the insecticidal properties of nucleopolyhedrovirus occlusion bodies (OBs) by compromising the integrity of the larval peritrophic matrix. However, at a low dose concentration (1 mg/L), flufenoxuron did not enhance the insecticidal activity of SeMNPV or SfMNPV in their respective host [144].

Sequential applications with *Bacillus thuringiensis* [145], Spinosad [29], or azadirachtin [146] have proven effective in reducing selection pressure and improving control in resistant populations. Metaflumizone retains its efficacy regardless of the order of application relative to nucleopolyhedrovirus. In case of *Spodoptera littoralis*, significant synergy has been reported between *Spodoptera littoralis* nucleopolyhedrovirus (SpliNPV) and azadirachtin, as well as SpliNPV and emamectin, particularly when the baculovirus is applied first [146]. Combinations with indoxacarb or emamectin benzoate extend the lifespan of infected larvae, favoring horizontal virus transmission. Moreover, SeMNPV combined with emamectin benzoate and chlorfenapyr has increased insecticidal efficacy and decreased resistance in *Spodoptera exigua* [29]. Synergy has also been observed between different baculovirus species. For example, *Spodoptera littoralis* nucleopolyhedrovirus (SpliNPV), which infects *Spodoptera littoralis*, and *Autographa californica* multiple nucleopolyhedrovirus (AcMNPV), used against *Spodoptera exigua*, when combined with metaflumizone or emamectin, increase mortality in *Spodoptera exigua* [146].

Another promising strategy is the encapsulation of SeMNPV OBs in modified calcium alginate microcapsules, which improves UV resistance and insecticidal efficacy, facilitating controlled-release applications [147]. This virus has also shown efficacy comparable to methomyl and permethrin in reducing *Spodoptera exigua* infestations in lettuce crops in California [148].

In field settings, the co-application of SfMNPV and spinosad has significantly reduced *S. frugiperda* incidence, particularly under sequential schemes or in combination with bio-stimulants such as biol-based products [24]. Spinosad does not impair viral replication when applied after viral inoculation. Likewise, neem acts as a repellent and growth regulator, prolonging the larval stage and promoting viral replication and horizontal dissemination [146]. Due to its slow action, chlorantraniliprole allows infected larvae to survive long enough to spread the virus, especially when applied at sublethal doses [29].

The combination of SfMNPV with spinetoram has been identified as one of the most effective strategies for reducing larval populations and damage, outperforming treatments involving emamectin benzoate and chlorantraniliprole [149]. Furthermore, applying SfMNPV at a lethal concentration along with a sublethal dose of spinetoram resulted in synergistic effects, achieving 86.66% larval mortality. Similar outcomes were found with lethal doses of NPV combined with sublethal concentrations of chlorantraniliprole, or emamectin benzoate [150].

#### 3.5.2. Antagonism Interactions

The combined application of baculoviruses with other bioinsecticides, such as spinosad, can result in complex interactions whose outcomes are not always favorable. While numerous studies have documented synergistic or additive effects that enhance the efficacy of biological control, it is equally important to recognize the potential for antagonistic interactions, which may critically reduce treatment success [151]. These adverse effects, far from being isolated cases, represent a significant risk to the sustainability of integrated pest management (IPM) strategies, as they can diminish viral infectivity, alter the physiological response of the pest, and compromise the overall effectiveness of biological agents. The nature and extent of these interactions are strongly influenced by factors such as the pest species, larval instar, and application doses, emphasizing the need for rigorous compatibility assessments before their use in the field [152].

In particular, spinosad, despite its reported synergistic effects when applied sequentially, has shown antagonistic interactions when co-applied or used at high concentrations, mainly due to the rapid paralysis it induces in larvae, which limits the ingestion of viral occlusion bodies and thus reduces viral infection efficiency [24,29]. Similar antagonistic outcomes have been observed in combinations of PxNPV with *Bacillus thuringiensis* strain LBIT-229, where both laboratory and field trials reported increased LC_50_ values and lower larval mortality compared to treatments with each agent applied individually [30].

These antagonistic interactions represent a critical challenge for the optimization of integrated pest management strategies, as they can substantially undermine the overall efficacy of biological control agents. The outcomes of such combinations are highly context-dependent and are influenced by various biological and environmental factors, including the pest species, larval developmental stage, and the application dose. While synergistic or additive interactions have been widely reported, such as in the combinations of nucleopolyhedroviruses with *Azadirachta indica* [153] and *Bacillus thuringiensis* [154], antagonistic effects have also been documented, notably in the interaction between NPV and imidacloprid [155].

These antagonistic outcomes are believed to result from physiological disturbances in the insect host, such as reduced feeding activity or alterations in midgut pH, both of which can impair viral infection and replication processes [156]. Such evidence highlights the intricate nature of bioinsecticide interactions and underscores the necessity for comprehensive compatibility assessments before their combined application in pest management programs. In the specific case of spinosad, ref. [157] demonstrated that both sublethal and lethal doses increased larval mortality and developmental delays, potentially enhancing the window of susceptibility to baculovirus infection. However, these beneficial effects may be counterbalanced if adverse physiological responses in the host hinder the successful establishment of viral infections. Therefore, while the combination of baculoviruses and spinosad holds considerable promise for enhancing pest control efficacy and delaying resistance evolution, careful evaluation of potential antagonistic effects is imperative to safeguard the long-term success and sustainability of biological control strategies for major agricultural pests [158].

For SeMNPV, co-application with deltamethrin can be counterproductive, as this insecticide may kill larvae before viral replication is complete [159]. Some pyrethroids have also been shown to directly inactivate OBs when tank mixed. Concurrent spinosad application may induce early paralysis, limiting viral ingestion, in contrast to the benefits observed in staggered treatments.

Low-dose applications of flufenoxuron failed to enhance OB production or larval mortality, suggesting that its effects are dose- and species-dependent [144]. Methoxyfenozide may induce premature molting that interrupts the viral cycle. If applied post-SeMNPV infection, it may reduce viral yield or delay mortality, leading to subadditive or antagonistic effects [29].

Finally, certain adjuvants in commercial formulations may degrade the viral capsid or alter pH levels, ultimately compromising viral viability [160].

The synergistic or antagonistic interactions between active substances and baculoviruses can be attributed to several hypotheses involving physiological, immunological, and biochemical effects in the insect host. Some compounds have been shown to cause damage to the midgut epithelium, thereby facilitating viral entry and dissemination. In contrast, others have been observed to promote the repair of the epithelium, which in turn restricts the infection. At the immunological level, certain substances may suppress the host’s immune response and enhance viral replication, whereas others activate defense mechanisms that hinder viral efficacy. Additional mechanisms have been proposed as well, including alterations in metabolic pathways, competition for cellular machinery, and changes in feeding behavior. These factors may influence infection efficiency depending on the specific combination evaluated. In this context, the optimization of dosage and the timing of application typically staggered by 24 to 48 h is critical to minimizing antagonistic effects, considering the properties of both the active compound and the baculovirus used, Table 3.

### 3.6. Examples of Baculovirus Resistance Emergence in Target Populations

Baculovirus represents a valuable alternative to chemical insecticides in controlling resistant pest populations. Although cases of resistance to these viral agents are considerably rarer and less pronounced than those seen with conventional insecticides, resistance evolution remains a legitimate concern. Abot et al. [171] demonstrated resistance in *Anticarsia gemmatalis* to *AgMNPV* under laboratory selection pressure, with notable increases in the median lethal concentration (LC_50_) within four generations. This resistance was more pronounced in Brazil, where the virus is endemic, compared to the United States, where its absence limited resistance evolution. Such findings highlight how environmental context shapes resistance dynamics.

Field conditions, however, may mitigate resistant establishment due to ecological complexity. Briese and Mende [172] reported that *Phthorimaea operculella* field populations in Australia were significantly less susceptible to a granulovirus than laboratory-reared counterparts. Endemicity, genetic variability, and the influx of susceptible individuals from untreated areas observed in Brazil’s AgMNPV-treated soybean systems can dilute resistant genotypes and reduce selection pressure. Unlike the constant and controlled conditions in laboratory assays, field selection intensity is modulated by population density, host age, climate, and viral heterogeneity.

Nevertheless, well-documented cases like the widespread resistance of *Cydia pomonella* to CpGV across Europe demonstrate that repeated use of a single viral strain can drive resistance, particularly when resistance is heritable and mechanistically linked to early-stage viral blockage [173]. Strategic interventions, including the rotation of CpGV variants (e.g., E2, I08, I12, S, and V15) and continuous susceptibility assessments, are essential to sustain viral efficacy.

Specific baculoviruses discussed in this study, PxGV, PxNPV, SeMNPV, and SfMNPV have shown varying resistance patterns. Resistance in diamondback moth larvae to PxGV increases with larval development [142,163], and susceptibility to SeMNPV similarly decreases with age in *Spodoptera exigua* [174]. Certain *S. exigua* cell clones harboring partial SeMNPV genomes exhibit resistance to homologous infections [175], and *Agrotis segetum* has shown resistance to SeMNPV despite genomic similarity to AsNPV [176]. Nutritional conditions also affect resistance levels; higher protein diets increase *S. exigua* tolerance to SeMNPV [177]. Additionally, immune-related factors like *S. exigua* peptidoglycan recognition proteins of the long-type class (SePGRP-LB) have been shown to reduce SeMNPV susceptibility through extracellular antiviral activity [178].

Conversely, SfMNPV exhibits no cross-resistance with chemical insecticides or Bt proteins [179], underscoring its compatibility with IPM. Furthermore, increasing evidence suggests that genotypically diverse baculovirus mixtures can delay resistance emergence and enhance viral adaptability, though potential fitness cost modulated by environmental and host factors must be considered [17]. These findings advocate for incorporating ecological and evolutionary principles into the design of sustainable, virus-based pest control strategies.

### 3.7. Limitations and Perspectives

The widespread use of synthetic chemical insecticides has led to the development of resistance in pest insects and pathogens. In addition to diminishing their efficacy, these compounds pose significant health risks, cause environmental degradation, and incur high economic costs. In response to these challenges, IPM has gained prominence as a sustainable approach that aligns ecological responsibility with crop protection.

This article reviews the scientific literature on the individual and combined use of baculoviruses with conventional chemical insecticides or bioinsecticides to control *Plutella xylostella*, *Spodoptera exigua*, and *Spodoptera frugiperda* in broccoli, tomato, and maize crops, respectively.

One of the main limitations identified in this study is the limited characterization of the vast diversity of baculovirus. Specifically, the baculoviruses examined, PxGV, PxNPV, SeMNPV, and SfMNPV, have been evaluated in relatively few studies, leaving significant opportunities to explore effective doses of both chemical and biological insecticides in combination with these baculoviruses and their target hosts.

Looking ahead, future research should aim to assess the efficacy of synergistic combinations of baculoviruses with sublethal doses of chemical insecticides versus bioinsecticides. These assessments should focus on treatment efficiency, specifically regarding pest reduction over time and suppression percentages. Such insights are crucial for achieving key goals of environmental preservation, food security, and protecting human health and non-target organisms, including beneficial insects and vertebrates.

Finally, the extrapolation of successful synergistic combinations to other cropping systems is inherently complex and demands a multidimensional perspective. Critical factors that influence this transferability include variations in host insect behavior, phenology, and immunocompetence, which can affect the optimal dose and formulation of the combination.

Equally important are the physicochemical properties of the plant surface, such as epicuticular waxes, phenolic compounds, and leaf pH, which may alter baculovirus stability and efficacy. These variables often necessitate a recalibration of application rates to sustain synergy interactions under the specific conditions of the new crop.

**Figure 2:**
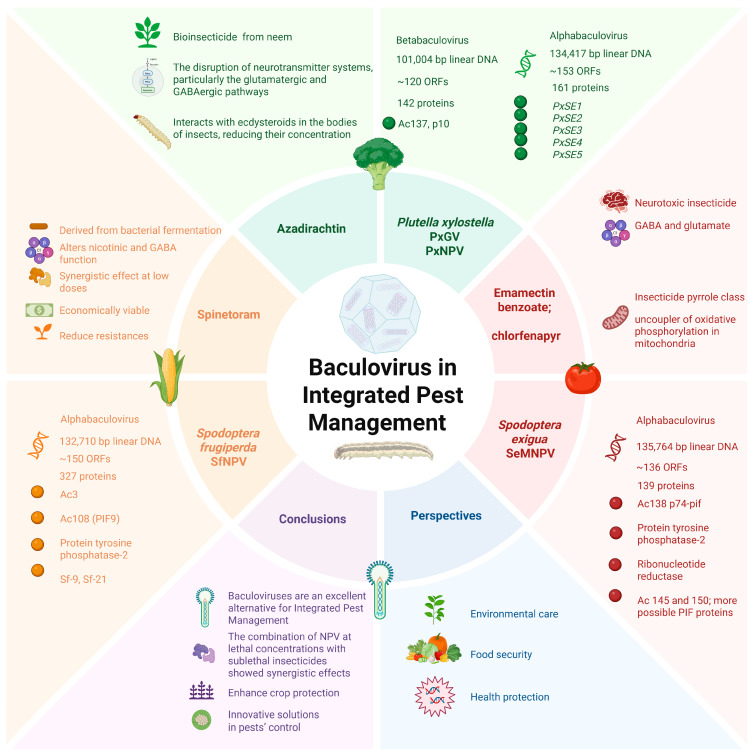
Baculovirus in integrated pest management. The use of baculoviruses for the control of *Plutella xylostella* in broccoli, *Spodoptera exigua* in tomato, and *Spodoptera frugiperda* in maize crops. This figure summarizes key aspects of various baculoviruses including genome size (in base pairs), the number of open reading frames (ORFs), proteins identified according to the UniProt database, and proteins that confer specificity to the respective pest. Additionally, it discusses the insecticides with which these baculovirus exhibit synergy. Bioinsecticides are proposed as an environmentally friendly alternative. The figure concludes with the main conclusion and perspectives.

In addition, agronomic practices associated with the target crop, such as prior pesticide exposure, fertilization regimes, and irrigation strategies, can influence the persistence and compatibility of virus insecticide formulations. Therefore, a thorough evaluation of the crop’s ecological and management context is essential to anticipate and mitigate potential antagonistic interactions, as seen in Figure 2.

## 4. Conclusions

Crop protection and pest control remain ongoing challenges for farmers worldwide. Chemical insecticides have facilitated pest management through a range of mechanisms of action; however, the increasing incidence of resistance development has compromised their long-term effectiveness. In this context, baculoviruses play a vital role in biological pest control, offering a sustainable and environmentally friendly alternative, particularly when conventional strategies lose efficacy due to resistance.

This study summarizes findings on the individual and combined use of PxGV, PxNPV, SeMNPV, and SfMNPV with either chemical or biological insecticides, highlighting cases of both synergistic and antagonistic interactions. Overall, evidence supports that the combination of a sublethal dose of a chemical insecticide with a lethal dose of baculovirus can result in synergistic effects. Moreover, the application of natural active compounds before virus administration, or the sequential application of fast-acting chemical insecticides 24 to 48 h after viral treatment, has shown enhanced efficacy.

The implementation of IPM programs can significantly reduce reliance on chemical pesticides and improve crop protection outcomes. Therefore, the combined use of genetically diverse baculoviruses with chemical or biological insecticides across different crops and target pests remains a promising avenue for research and development in resistance management. This approach not only addresses current ecological concerns but also lays the groundwork for innovative solutions in sustainable pest control.

## Figures and Tables

**Table 1:** Steps associated with planning data collection for this study.

Phases	Description
1	Definition of the study theme and guiding question (PICO)
2	Establishment of selection criteria (inclusion and exclusion of articles)
3	Selection of databases and descriptors for literature access
4	Data collection
5	Analysis of results

Source: Adapted from [31].

**Table 2:** Chemical and biological strategies for the control of *P. xylostella*, *S. exigua*, and *S. frugiperda* in broccoli, tomato, and maize crops, respectively.

Chemical Family *	Molecular Target	Active Compound (Treatment)	Crop	Effectiveness Plague	Effective Physiology in Plagues	Effectiveness (%)	Resistance	Non-target Insect Toxicity	Human Toxicity	References
1A Carbamates	1 Acetylcholinesterase (AChE) inhibitors	Carbosulfan	Broccoli	*P. xylostella*,	Nerve and Muscle	70–80	Yes	High	High	[50,51,52,53]
		Carbofuran	Tomato	*S. exigua*	Nerve and Muscle	80–90	Yes	High	High	[54,55,56,57]
		Methomyl	Tomato, Maize	*S. exigua*, *S. frugiperda*	Nerve and Muscle	80–90	Yes	High	High	[58,59,60,61]
1B Organophosphates		Chlorpyrifosmethyl	Tomato	*S. exigua*	Nerve and Muscle	Up to 90	Yes	High	High	[62,63,64,65]
		Methamidophos	Tomato	*S. exigua*	Nerve and Muscle	85–90	Yes	High	High	[66]
		Acephate	Tomato	*S. exigua*	Nerve and Muscle	85–90	Yes	High	High	[67]
3A Pyrethroids	3 Sodium channel modulators	Permethrin	Broccoli, Tomato	*P. xylostella*, *S. exigua*	Nerve and Muscle	70–85	Yes	High	High	[11,68,69,70,71,72,73,74]
		Bifenthrin	Tomato	*S. exigua*	Nerve and Muscle	85–95	Yes	High	High	[75,76]
		Cypermethrin	Tomato	*S. exigua*	Nerve and Muscle	85–95	Yes	High	High	[77,78,79,80]
		Gamma Cyhalothrin	Tomato	*S. exigua*	Nerve and Muscle	85–95	Yes	High	High	[81,82,83]
4A Neonicotinoids	4 Nicotinic acetylcholine receptor (nAChR) competitive modulators	Neonicotinoids	Broccoli	*P. xylostella*	Nerve and Muscle	75–85	Yes	Low	Low	[84,85,86,87,88]
5 Spinosyns	5 Nicotinic acetylcholine receptor (nAChR) allosteric modulators site I	Spinosad	Broccoli	*P. xylostella*	Nerve and Muscle	80–90	Yes	Low	Low	[89,90,91,92,93,94]
6 Avermectins and Milbemycins	6 Glutamate-gated chloride channel (GluCl) allosteric modulators	Emamectin benzoate	Maize Tomato	*S. frugiperda S. exigua*	Nerve and Muscle	90	Yes	High	High	[95,96]
8C Fluorides	8 Miscellaneous non-specific (multisite) inhibitors	Cryolite	Tomato	*S. exigua*	Unknown or Non-specific	70–80	Yes	Low	Low	[97,98]
11A *Bacillus thuringiensis*	11 Microbial disruptors of insect midgut membranes	*Bacillus thuringiensis*	Tomato	*S. exigua*	Midgut	70–90	Yes	High	Low	[47,48]
13 Pyrroles, Dinitrophenols, Sulfluramid	13 Uncouplers of oxidative phosphorylation via disruption of proton gradient	Chlorfenapyr	Maize Tomato	*S. frugiperda, S. exigua*	Respiration	85.18	Yes	High	High	[99,100,101]
15 Benzoylureas	15 Inhibitors of chitin biosynthesis affecting CHS1	Novaluron	Tomato	*S. exigua*	Growth and Development	70–85	Yes	High	Low	[102,103]
22 Oxadiazines	22 Voltage-dependent sodium channel blockers	Indoxacarb	Maize	*S. frugiperda*	Nerve and Muscle	94.23	Yes	High	High	[10,104,105]
28 Diamides	28 Ryanodine receptor modulators	Chlorantraniliprole	Maize	*S. frugiperda*	Nerve and Muscle	90	Yes	High	Moderate	[106,107,108]
31 Granuloviruses and Nucleopolyhedroiruses	31 Baculoviruses	Baculovirus	Broccoli	*P. xylostella*	Midgut	60–75	No	None	None	[30,109,110,111]
Unclassified	Unknown or uncertain mode of action	Azadirachtin	Tomato	*S. exigua*	Unknown or Non-specific	70–85	Yes	Low	Low	[5,112,113]

* Classification according to R4P (https://www.r4p-inra.fr/en/ppps-classification/ accessed on 20 May 2025).

**Table 3:** Synergistic and antagonistic combinations of GV and NPV with insecticides.

Active Compound	*Plutella xylostella* nucleopolyhedrovirus (PxGV) * (PxNPV)		*Spodoptera exigua* nucleopolyhedrovirus (SeMNPV)		*Spodoptera frugiperda* nucleopolyhedrovirus (SfMNPV)		References
	Synergy	Antagonism	Synergy	Antagonism	Synergy	Antagonism	
**Methomyl**	-	-	Yes	-	-	-	[143]
**Chlorpyrifos methyl**	-	-	-	-	Yes	-	[161]
**Deltamethrin**	-	-	-	Yes	-	-	[159]
**Lambda Cyhalothrin**	-	-	-	-	Yes	-	[161]
**Spinosad**	Yes	Yes	Yes	Yes	Yes	Yes	[24,29]
**Spinetoram**	-	-	Yes		Yes	-	[29,149]
**Emamectin benzoate**	-	-	Yes		Yes	-	[29,150]
***Bacillus thuringiensis***	Yes *	Yes	Yes	-	-	-	[30,145,162]
***Beauveria bassiana***	Yes *	-	-	-	-	-	[142]
***Metarhizium rileyi***	-	-	-	-	Yes	-	[163]
**extract of *Vitex trifolia***	-	-	Yes	-	-	-	[164]
**Chlorfenapyr**	-	-	Yes	-	-	-	[29]
**Flufenoxuron**	-	-	-	Yes	-	Yes	[144]
**Novaluron**	Yes	-	-	-	Yes	-	[161]
**Tebufenozide**	Yes	-	Yes	-	-	-	[159]
**Methoxyfenozide**	-	-	-	Yes	-	-	[29]
**Indoxacarb**	Yes *	-	Yes	-	-	-	[29,142]
**Chlorantraniliprole**	Yes	-	Yes	-	Yes	-	[29,150]
**SpliNPV, AcMNPV, or LdMNPV**	Yes	-	Yes	-	Yes	-	[140,165,166,167]
**Azadirachtin**	Yes *	-	Yes	-	Yes	-	[141,146]
**Fipronil**	Yes *	-	-	-	-	-	[142]
**Methoprene**	-	-	Yes	-	-	-	[168]
**Stilbene derivatives optical brighteners**	-	-	Yes	-	-	-	[169,170]
**Microencapsulation Formulations**	-	-	Yes	-	Yes	-	[147,161]
**Aggressive surfactants or solvents**	-	-	-	-	-	Yes	[160]

(*) Indicates instances where the active compound specifically interacts with *Plutella xylostella* granulovirus.

## Abbreviations

The following abbreviations are used in this manuscript:

PxNPV	*Plutella xylostella* nucleopolyhedrovirus
PxGV	*Plutella xylostella* granulovirus
SeMNPV	*Spodoptera exigua* multiple nucleopolyhedrovirus
SfMNPV	*Spodoptera frugiperda* nucleopolyhedrovirus
NPV	Nucleopolyhedrovirus
GV	Granulovirus
PICO	Problem, Intervention, Comparison, Outcome
IPM	Integrated Pest Management
ODV	Occlusion-Derived Viruses
PIF	*Per os* Infectivity Factor
GP64	Glycoprotein 64
kDa	Kilo daltons
ORF	Open Reading Frames
bp	Base pairs
SINEs	Short Interspersed Nuclear Elements
tRNA	Transfer RNA
GDP	Gross Domestic Product
PTP2	Protein Tyrosine Phosphatase-2
GABA	Gamma-Aminobutyric Acid
ATP	Adenosine Triphosphate
DNA	Deoxynucleotide Triphosphate
